# Water radiolysis by low-energy carbon projectiles from first-principles molecular dynamics

**DOI:** 10.1371/journal.pone.0171820

**Published:** 2017-03-07

**Authors:** Jorge Kohanoff, Emilio Artacho

**Affiliations:** 1 Atomistic Simulation Centre, Queen’s University Belfast, Belfast BT7 1NN, Northern Ireland, United Kingdom; 2 Department of Earth Sciences, University of Cambridge, Cambridge CB2 3EQ, United Kingdom; 3 Theory of Condensed Matter, Cavendish Laboratory, University of Cambridge, Cambridge CB3 0HE, United Kingdom; 4 CIC Nanogune and DIPC, Tolosa Hiribidea 76, 20018 San Sebastián, Spain; 5 Basque Foundation for Science Ikerbasque, 48013 Bilbao, Spain; University of Lincoln, UNITED KINGDOM

## Abstract

Water radiolysis by low-energy carbon projectiles is studied by first-principles molecular dynamics. Carbon projectiles of kinetic energies between 175 eV and 2.8 keV are shot across liquid water. Apart from translational, rotational and vibrational excitation, they produce water dissociation. The most abundant products are H and OH fragments. We find that the maximum spatial production of radiolysis products, not only occurs at low velocities, but also well below the maximum of energy deposition, reaching one H every 5 Å at the lowest speed studied (1 Bohr/fs), dissociative collisions being more significant at low velocity while the amount of energy required to dissociate water is constant and much smaller than the projectile’s energy. A substantial fraction of the energy transferred to fragments, especially for high velocity projectiles, is in the form of kinetic energy, such fragments becoming secondary projectiles themselves. High velocity projectiles give rise to well-defined binary collisions, which should be amenable to binary approximations. This is not the case for lower velocities, where multiple collision events are observed. H secondary projectiles tend to move as radicals at high velocity, as cations when slower. We observe the generation of new species such as hydrogen peroxide and formic acid. The former occurs when an O radical created in the collision process attacks a water molecule at the O site. The latter when the C projectile is completely stopped and reacts with two water molecules.

## Introduction

Water dissociation and the formation of other molecules by the action of radiation is one of the most important radiolytic processes, and has been studied for over a century by many authors. [1] While the main interest in the subject is traditionally related to biological implications, [1, 2] and to nuclear reactor design, [3] it recently came into focus also within the energy context, due to the possibility of generating hydrogen at low cost. [4] We will focus this study on ionic projectiles, and will not consider electromagnetic radiation. The two main natural occurrences of ions are: in space in the form of cosmic rays (mostly protons, *α*-particles and electrons), and as products of radioactive decay in radionuclides. However, high-energy ions can also be produced in accelerators and used as radio-therapeutic tools (hadron-therapy). In either case, it is of major interest to understand, at the microscopic level, how do protons, *α*-particles and heavier ions like C^+*q*^ interact and split water or, in a biological context, produce reactive fragments that induce biological end-point effects such as DNA damage.

Most of these particles are very energetic (keV to MeV). When water is exposed to radiation of this nature the main effect is ionization, whereby electrons in the water orbitals are removed. The result is a characteristic distribution of secondary electrons whose kinetic energy peaks at low kinetic energies and then decreases monotonically. [5, 6] Other collision channels such as ion-molecule direct impact have exceedingly small cross sections in this regime, and can be ignored. The ionization regime can be described quite well in terms of binary collisions with individual water molecules (gas phase) where the electronic structure is corrected for the influence of the environment (condensed phase). [7] The information on scattering cross sections can then be used to study radiation tracks via Monte Carlo simulations. [7, 8]

As ions travel through the medium ionizing the water, they gradually lose their energy. Initially, the ionization cross section is small, but when their velocity approaches that of the electrons in the water orbitals, a resonance phenomenon takes place and a peak in the absorbed dose is observed (Bragg peak), which for carbon corresponds to a state of charge approximately C^3+^. [9] Beyond the Bragg peak, the ionization cross section and the velocity of the ions rapidly decrease while the ions capture additional electrons. Below a certain threshold, ionic projectiles do not have enough kinetic energy to ionize water. The electronic excitation channel remains open, but only briefly. Water is an electronic wide gap insulator like LiF, for which the existence of a projectile-velocity threshold for electronic excitation has been shown [10] (and partly understood. [11, 12]) to be between 0.1 and 0.2 atomic units of velocity. For carbon projectiles and using what learned for LiF, the electronic excitation channel should essentially close at energies of the order of 4 keV. Below this, the collision process should be predominantly adiabatic, meaning that electrons remain in the instantaneous ground state corresponding to the nuclear configuration.

This regime is extremely interesting for various reasons. In comparison with the ionization regime, it has always been assumed that low-energy ions produce comparatively little (if any) damage but, in fact, it remains largely unexplored. Within the radio-therapeutical context, where the energy of the incoming ions is adjusted to optimize the energy deposition, this region occurs beyond the Bragg peak. In the adiabatic regime, energy is transferred directly into translational, rotational and vibrational degrees of freedom of the target molecules. If a sufficient amount of energy is deposited into vibrations, then water molecules can dissociate thus originating various fragments like OH^−^, H^+^ and O^−^ ions, and OH^•^, H^•^ and O^•^ radicals. These, in turn, are reactive species that tend to either recombine, if the fragments have not travelled too far away from each other, or to associate with other fragments and with water molecules, thus giving rise to more complex entities such as hydrogen peroxide (H_2_O_2_) and ${\text{HO}}_{2}^{•}$. Within this regime, the binary collision hypothesis becomes questionable, and the use of gas phase calculated or measured scattering cross sections requires careful validation.

In this work we study the collision and chemical processes that occur when a carbon projectile traverses a slab of liquid water, by first-principles molecular dynamics simulations, within the adiabatic regime. We show that the largest density of damage occurs at projectile energies well below the peak in energy deposition. This is consistent with experimental findings of low-velocity C^+^ ions impinging on DNA plasmid samples. [2] The explanation is that at higher velocities collisions are more infrequent, but most of the transferred energy goes into kinetic energy of the collision products. Therefore, the amount of energy stored into locally reactive species is approximately constant, but at lower velocities it is spatially more densely distributed.

## Methods

The work is based on first-principles molecular-dynamics simulations (FPMD). These have been carried out with the Siesta code, [13] which is based on density functional theory and pseudopotentials, and uses numerical pseudoatomic orbitals as a basis set. A reasonable description of hydrogen bonding in water [14] is given by the generalized gradient approximation as proposed in [15] (GGA-PBE). The pseudopotentials and basis functions have been validated previously in a study of bulk liquid water. [14] A previous study on the behaviour of pseudopotentials for binary collisions [16] supports the pseudopotentials used here for the scale of energies and distances attained.

A bulk sample of 128 water molecules was equilibrated with FPMD at a temperature of 300 K over 10 ps in a cubic box of side 15.736 Å, under periodic boundary conditions. For practical purposes a water slab was generated, which allows for a better definition of the initial and final states of the sample and the projectile, as well as of the energy loss of the latter. It should be remarked, however, that it is the bulk behaviour what is explored here, not the thin-film or surface effects. For that purpose, the bulk liquid box was expanded to twice that length along the *x*-direction without modifying the atomic positions, the extra space playing the role of a vacuum slab. The water slab so created was further equilibrated with FPMD for 0.5 ps, which is sufficient time for the molecules to accommodate in the short range, but intentionally insufficient for larger range relaxations (surface layering etc). Two snapshots were extracted from the equilibrated run, and the shooting of a carbon projectile through the water slab was simulated for a set of 36 different initial conditions for each snapshot and initial velocity, thus totalling 72 trajectories per velocity. The projectile was shot from locations in vacuum distributed in a uniform square grid in the *yz*-plane, covering the whole surface area of the slab. The initial velocity of the projectile was chosen perpendicular to the slab, i.e. along the *x*-direction. The overall charge of the system was set to +1*e*, and was compensated with a uniform neutralizing background as required for extended periodic systems (C^+^ is the relevant charge state at low velocities [2]). We studied four different initial velocities, 0.025, 0.05, 0.075 and 0.1 a.u. corresponding to kinetic energies 175 eV, 700 eV, 1.6 keV and 2.8 keV, respectively. The latter is close, yet below the estimated limit of validity of the adiabatic hypothesis. The Newtonian equations of motion for the nuclei were integrated using a velocity Verlet algorithm with a time step of 0.1 fs.

## Results and discussion

**Fig 1:**
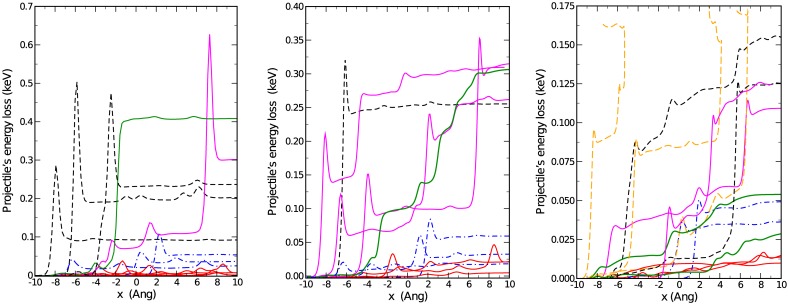
Energy lost by the projectile to the medium as a function of the distance traversed in the water slab. Left panel: Initial energy 2.8 keV. Thin solid (red) lines represent non-dissociative trajectories. Dashed (black) lines involve the complete dissociation of a single molecule, and dot-dashed (blue) lines are for single H dissociation from a water molecule. The two thick solid lines represent rare events of multiple, sequential dissociation (magenta) and large kinetic energy transfer to a single H (green). The pronounced peaks occur when the projectile reaches very close to the target, but this energy is then partially restored. Middle panel: Initial energy is 0.7 keV. Lines and colors as in left panel, except for the thick solid (green) line, which corresponds to a trajectory where the projectile transfers energy continually to the medium. Such cases cannot be described in terms of binary collisions. Right panel: Initial energy is 0.175 keV. Lines and colors as middle panel. Long-dashed orange lines represent trajectories where the projectile is stopped completely within the water slab. The peaks just before each collision are due to the projectile experiencing the repulsion of a target atom at close proximity.

We first analyze the various types of trajectories and collision events by plotting the evolution of the energy loss of the projectile to the water for some selected, representative simulations. Fig 1 shows that for an initial kinetic energy of 2.8 keV, collisions with the water molecules are not very frequent, thus suggesting that the cross section is relatively small. Nevertheless, for small impact parameters, collisions are quite dramatic, invariably leading to the dissociation of the water molecule, either singly (C+H_2_O→C+OH+H) or doubly (C+H_2_O→C+O+2H). The energy loss to the fragments is quite significant, between 25 and 50 eV for single dissociation events (dot-dashed lines in Fig 1), and 100 to 150 eV for complete dissociation (dashed lines). Within our GGA-PBE and also experimentally, [17] the dissociation energy of the first H-atom in the water molecule is about 5 eV. The second H dissociates for an extra 4.4 eV. [17] These energies are always small compared to the total energy transferred, amounting to a 5–10% at the most. The remainder is transferred as kinetic energy of the fragments, which are thus produced as hyperthermal species. [18] There are also many trajectories where the projectile traverses the sample without impacting any water molecule, and losing only a small fraction of its energy into non-dissociative channels such as vibrational excitation. An important observation is that, at such large kinetic energies, the projectile proceeds via a succession of individual collision steps, thus justifying the binary collision model. Such individual collisions are evident in the sharp peaks exhibited by the curves in Fig 1. There, the projectile experiences a collision with one of the atoms in the target. This collision has an inelastic and an elastic component, the latter being responsible for the peak. Kinetic energy is temporarily converted into potential energy, and then quickly recovered.

Simulations with initial energy of 1.6 keV are not qualitatively very different from those at 2.8 keV. Therefore, we next analyze trajectories generated for an initial kinetic energy of 700 eV (middle panel in Fig 1). Now multiple collision trajectories are much more frequent. The energy transfers are slightly, but not significantly smaller, i.e. around 100 eV for dissociative events. This is a larger fraction of the initial energy, but not large enough to stop completely the projectile. There is a noticeable increase in the amount of secondary particles generated by dissociation, due to the enhanced cross section at lower velocities. By inspecting the trajectories it can be observed that in this regime the projectile has time to *drag* the particles, thus giving rise to less dramatic dissociation events where the O-H stretching vibrational mode is excited beyond the point of no return (see S1–S6 Videos in the Supporting Information). In some cases this proton joins another molecule forming H_3_O^+^ while the remaining OH^−^ reacts with a water molecule to form H_3_O_2_^−^. One of the trajectories depicted in dot-dashed (blue) lines corresponds precisely to the creation of this species, at an energetic cost of 16 eV for the projectile.

An even more interesting feature emerging at this velocity is the appearance of trajectories where the energy loss is not by steps, but rather in an almost continuous way by dissociating water molecules and exciting vibrational modes. This is represented by the thick (green) solid line. This class of trajectories cannot be satisfactorily described in terms of binary collisions, thus signalling the breakdown of binary collision models.

In the right panel we show the evolution of the projectile’s kinetic energy for an even lower initial energy of 175 eV. Now the majority of the trajectories presents a continuous energy loss to vibrations and rotations which, due to the scale of the energy transfers, becomes more evident. A new type of trajectories appears at this velocity, where the projectile is completely stopped and thermalized in the medium (long-dashed orange lines). By inspecting these trajectories, it turns out that the energy transfer in dissociative collision events is still quite large, up to 100 eV (see dashed black and orange lines). Therefore, after two or three such events (orange lines), the projectile has exhausted all its kinetic energy and stops.

As before, the energy to dissociate the water molecules is a small fraction, and most of the kinetic energy is transferred to the hyperthermal fragments. In fact, in some trajectories the C is stopped, transferring its energy to an O atom or an OH group, which then assumes the role of the projectile. Other types of events observed are the abstraction of a H-atom by the projectile to form a travelling C-H group (see S1–S6 Videos in the Supporting Information). Here we restrict ourselves to an inventory of a variety of events that have been observed in the present simulations. We do not attempt to estimate the probability of occurrence of such events, which would require the careful evaluation of free energies and integration over impact parameter and water orientations, apart from statistical averaging.

It is important to remark that, since the dissociation energy is small compared to the energy transfer, the generation of secondary particles is not hindered at low energies. On the contrary, it appears enhanced with respect to the previous cases. The difference is that the H, O or OH fragments have lower kinetic energies, and are thus more prone to stop and react with other water molecules or fragments. In fact, in some cases we have observed the formation of new chemical species such as hydrogen peroxide H_2_O_2_, which is known to be one of the most common products in irradiated water within the ionization regime. [1] Interestingly, this phenomenon occurs here via the attack of a water molecule by an oxygen radical arising from a fully dissociating collision, unlike the conventionally accepted mechanism of the reaction between two OH radicals. [1]

**Fig 2:**
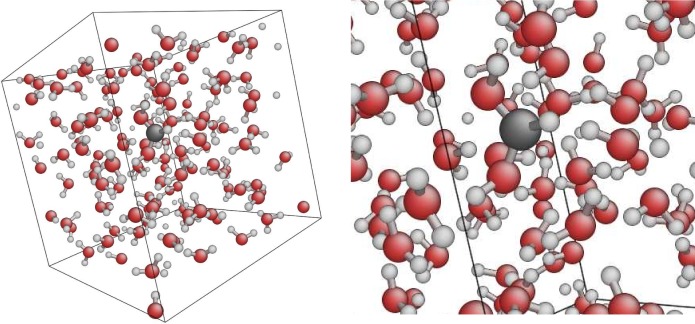
The formation of an organic chemical species by reacting carbon with water in the condensed phase. In this case the species is dihydroxymethyl radical (closely related to formic acid), which was formed during a first-principles MD simulation.

In order to study the situation after a C projectile has stopped, we have conducted simulations of a C^+^ ion in bulk water (128 molecules in a cubic box without vacuum). Here we have observed the formation of organic molecules such as the dihydroxymethyl radical ${\text{HCO(OH)}}_{2}^{•}$, where the carbon impurity has reacted with two water molecules, as shown in Fig 2. The formation of this species has also been observed in a recent study of irradiation of water clusters at 30 K, within the astrochemical context. [19]

**Fig 3:**
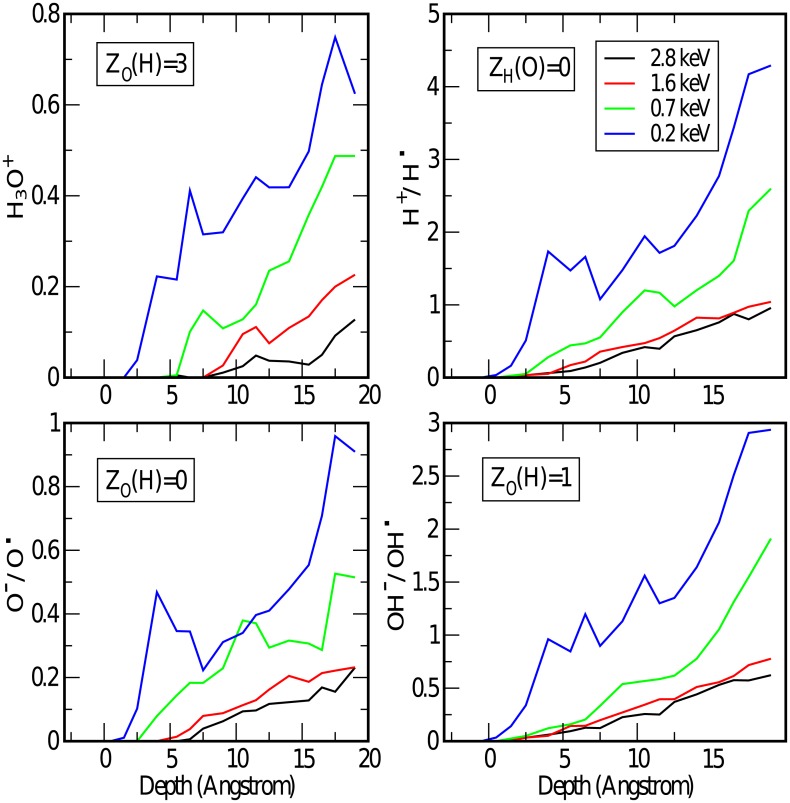
Evolution of the average number of secondary species generated during the passage of the projectile through the water slab, as a function of the spatial coordinate of the projectile. Upper left panel shows O-atoms coordinated to 3 H, lower left is for isolated O-atoms, upper right for isolated H-atoms, and lower right for O-atoms coordinated to 1 H-atom. The latter represents OH groups and also the less frequent H_2_O_2_ species. Colors correspond to the various initial conditions: 2.8 keV (black), 1.6 keV (red), 0.7 keV (green), and 0.17 keV (blue).

To analyze the distribution of species generated by the projectile, we have monitored the coordination number of the O and H-atoms along the track (see Fig 3) by considering only atoms within a specified distance cutoff, which we set to 1.2 Å, except for H-H pairs which we set to 0.8 Å. Below, *Z*_*X*_(*Y*) indicates the number of *Y*-atoms coordinated to an *X*-atom.Thus, *Z*_*O*_(*Y*) = 0 for all *Y* implies an isolated oxygen atom or ion (lower left panel of Fig 3), *Z*_*O*_(*H*) = 1 indicates a hydroxyl anion or radical if *Z*_*O*_(*O*) = 0, or hydrogen peroxide if *Z*_*O*_(*O*) = 1 (lower right panel). If *Z*_*O*_(*H*) = 2 we have the usual water molecule (not shown), while *Z*_*O*_(*H*) = 3 represent hydronium ions, H_3_O^+^ (upper left panel). In the case of H-atoms, *Z*_*H*_(*Y*) = 0 for all *Y* implies hydrogen atoms or protons (upper right panel), while *Z*_*H*_(*H*) = 1 is for H_2_ molecules (not shown). *Z*_*H*_(*O*) = 1 can correspond to hydroxyl or hydronium groups, and *Z*_*H*_(*O*) = 2 suggests ${\text{H}}_{5}{\text{O}}_{2}^{+}$ groups (not shown). The quantities reported are the result of averaging over the 72 trajectories. The concentration of water fragments obtained is very substantially larger than the one observed at room conditions (∼ 10^−7^M at neutral pH). Water splitting processes (mostly into H^+^ and OH^−^) are also induced by high temperatures and pressures [20, 21], although a substantial splitting (∼ 1 mol %) is not observed below 2000 K and 12 GPa.

Interestingly, the density of secondary fragments generated along the track *increases* with decreasing velocity of the projectile. This is due to a resonant behavior of low-energy projectiles with molecular vibrations and rotations. At high velocities vibrations do not have the time to react to the passage of the projectile. Collisions are effective only for small impact parameters, being dissociation a secondary effect of the energy transfer. The spatial rate of fragment generation increases for slower projectiles up to a point in which dissociation events become less viable due to the small kinetic energies involved. The two most abundant fragments are H and OH groups. At the lowest energy studied here, the former are generated at a rate of one every 5 Å, while for OH it is one every 7 Å, the difference being due to the double dissociation events. This means that the projectile dissociates basically every molecule that finds in its way. This rate decreases with increasing energy, down to one H every 20 Å and one OH every 30 Å at 2.8 keV.

Hyperthermal fragments become secondary projectiles that originate secondary collision cascades, rather than thermalizing with the medium and locally heating the water. We have analyzed some of these trajectories. Interestingly, while the OH and O fragments rapidly experience other collisions, the mean free path for the H-atoms is much longer and they travel almost freely through the water film. Hence, much larger simulation boxes would be required to study radiolysis by protons or *α*-particles.

A very important quantity is the charge state of the projectile and the secondary fragments. It determines the strength of the interaction with the medium, and as such it is a crucial ingredient to compute cross sections in binary collisions. In addition, the charge state determines the reactivity of low energy fragments, and thus their damaging power, e.g. it is not the same an OH^•^ radical than a OH^−^ hydroxyl anion, or a proton and a hydrogen atom. In particular, neutrals tend to diffuse faster and longer than charged species.

The coordination numbers presented above do not carry information about the charge state. Therefore, we have to resort to some other method. Although the charge state of an atom or molecule in the condensed phase is not a well-defined quantity, Mulliken populations are a useful quantity to look at. The ambiguities intrinsic to any population (and to Mulliken’s) are small when calculating the population of a whole molecule, radical or ion. We have done this especially for the products of dissociative collision events, to understand whether the H and O atoms leave as neutral or charged species.

It is important to remark that all the processes considered here are adiabatic, in the sense that the charge is the result of the self-consistent determination of the charge density at each nuclear configuration. A hydrogen atom in a water molecule will exhibit a Mulliken charge of about 0.7, while a neutral hydrogen will carry a charge of 1 and a proton a zero charge. Since in the condensed phase a hydrogen atom can use the basis functions of other neighboring atoms, the charge will be generally smaller than 1. We observe that, when hyperthermal H or O atoms leave the slab, they do so as neutral atoms. However, when they remain in the slab, we observe the trend that hyperthermal hydrogens generally move as neutral atoms (with populations between 0.9 and 1 electron), while slow ones tend to move as protons (with populations smaller than 0.4 electrons, which account for the contribution of the H basis orbitals to the description of the electrons in close-by molecules).

**Fig 4:**
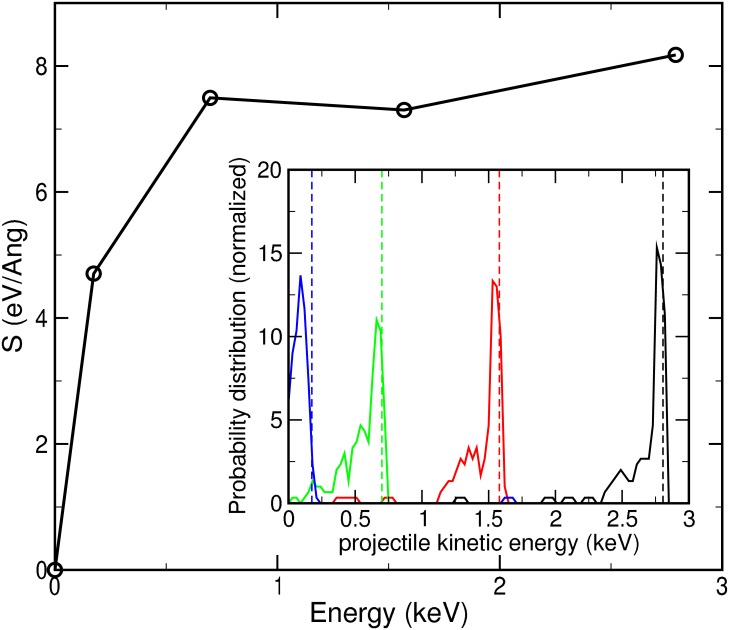
Nuclear stopping power as a function of projectile kinetic energy. The inset shows the distribution of final kinetic energies of the projectile for the various initial conditions: *v*_0_ = 0.1 a.u. (black), *v*_0_ = 0.075 a.u. (red), *v*_0_ = 0.05 a.u. (green), and *v*_0_ = 0.025 a.u. (blue). Vertical dashed lines indicate the initial kinetic energy.

72 trajectories per velocity value may not be sufficient to obtain good statistics for some properties. Nevertheless, there are some averaged quantities that are meaningful as qualitative indicators of the general behavior of the system. We first show the distribution of final kinetic energies of the projectile (inset to Fig 4). This has been calculated by monitoring its velocity once it has passed through the water slab and no more collision events occur, and collecting them in a histogram. Consistently with the previous observation that the magnitude of the energy transfer does not depend strongly on the energy of the projectile, the width of the distribution is practically energy-independent, except for the lowest energy where some projectiles are completely stopped. This information can be used to estimate the nuclear stopping power as the ratio between the energy deposited along the trajectory and the width of the slab. This is an estimate of a differential quantity averaged over a finite-width slab, which carries additional statistical uncertainties. Although it is not particularly accurate, it is qualitatively useful and we show it in Fig 4. It is tempting to conclude that there is a maximum in stopping power for energies around 2-3 keV, but statistical errors preclude a robust interpretation.

The rationale for such a maximum is that, qualitatively, the stopping power is determined by the cross section for the interaction of the projectile with individual molecules and the energy transferred in those collisions. While the former decreases with increasing energy, the latter increases but at a lower rate. Theoretical expressions for the location of this maximum do exist, [22] but they require the knowledge of the charge state of both projectile and target, and are not directly applicable to molecular targets. A crude estimate using available theories gives a value lower than the one observed here. [23] It should be emphasized that a more precise and statistically better defined nuclear stopping power would need substantial extra computation, which is beyond the scope of this paper, and could be the basis of future work. Similarly, further work would be interesting using higher levels of theory, whenever affordable. In particular, the introduction of self-interaction corrections (with spin polarization) would allow a better description of radical species, when exploring the processes in a finer energy scale, in addition to the explicit consideration of possible non-adiabatic effects. The main conclusions of this work, however, given the scales involved, are well supported by the level of theory employed.

## Conclusions

To summarize, the first-principles molecular-dynamics simulations presented for water radiolysis due to low-energy C bombardment, show high yields of H an OH fragments that act as secondary projectiles, especially for the higher velocities explored. They are however more abundantly produced for lower velocities, in multiple collisions that cannot be described in a binary framework. H secondary projectiles tend to move as neutral atoms (radicals) at the higher velocities observed, while it tends to move as a cation when slower. New species are seen to be produced, such as hydrogen peroxide and formic acid. The former occurs when an O radical created in the collision process attacks a water molecule. The latter when the C projectile is completely stopped and reacts with two water molecules.

## Supporting information

S1 Video C^+^ ion impinging on water with a kinetic energy of 175 eV. The different colors indicate different chemical species. There are six video files associated to this paper, corresponding to three different initial velocities of the C^+^ cation. All of them from the same initial conditions, except the magnitude of the velocity. They all correspond to initial position 29 within the 72 trajectories per velocity.
(MPG)

S2 Video C^+^ ion impinging on water with a kinetic energy of 175 eV. The different colors indicate different coordination.
(MPG)

S3 Video C^+^ ion impinging on water with a kinetic energy of 700 eV. The different colors indicate different chemical species.
(MPG)

S4 Video C^+^ ion impinging on water with a kinetic energy of 700 eV. The different colors indicate different coordination.
(MPG)

S5 Video C^+^ ion impinging on water with a kinetic energy of 2.8 keV. The different colors indicate different chemical species.
(MPG)

S6 Video C^+^ ion impinging on water with a kinetic energy of 2.8 keV. The different colors indicate different coordination.
(MPG)
